# Evaluating the quality and coverage of post-abortion care in Zimbabwe: a cross-sectional study with a census of health facilities

**DOI:** 10.1186/s12913-020-05110-y

**Published:** 2020-03-24

**Authors:** Taylor Riley, Mugove G. Madziyire, Onikepe Owolabi, Elizabeth A. Sully, Tsungai Chipato

**Affiliations:** 1 Guttmacher Institute, 125 Maiden Lane Suite 7, New York, NY 10038 USA; 2 Clinical Trials Research Centre (UZCHS-CTRC), University of Zimbabwe College of Health Science, 15 Phillips Road, Belgravia, Harare, Zimbabwe

**Keywords:** Post-abortion care, Quality of care, Signal functions, Health system, Abortion complications

## Abstract

**Background:**

An estimated 65,000 abortions occurred in Zimbabwe in 2016, and 40 % resulted in complications that required treatment. Quality post-abortion care (PAC) services are essential to treat abortion complications and prevent future unintended pregnancies, and there have been recent national efforts to improve PAC provision. This study evaluates two components of quality of care: structural quality, using PAC signal functions, a monitoring framework of key life-saving interventions that treat abortion complications; and process quality, which examines the standards of care provided to PAC patients.

**Methods:**

We utilized a 2016 national census of health facilities in Zimbabwe with PAC capacity (*n* = 227) and a prospective, facility-based 28-day survey of women seeking PAC in a nationally representative sample of those facilities (*n* = 1002 PAC patients at 127 facilities). PAC signal functions, which are the critical services in the management of abortion complications, were used to classify facilities as having the capability to provide basic or comprehensive care. All facilities were expected to provide basic care, and referral-level facilities were designed to provide comprehensive care. We also assessed population coverage of PAC services based on the WHO recommendation for obstetric services of 5 facilities per 500,000 residents.

**Results:**

We found critical gaps in the availability of PAC services; only 21% of facilities had basic PAC capability and 10% of referral facilities had comprehensive capability. For process quality, only one-fourth (25%) of PAC patients were treated with the appropriate medical procedure. The health system had only 41% of the basic PAC facilities recommended for the needs of Zimbabwe’s population, and 55% of the recommended comprehensive PAC facilities.

**Conclusion:**

This is the first national assessment of the Zimbabwean health system’s coverage and quality of PAC services. These findings highlight the large gaps in the availability and distribution of facilities with basic and comprehensive PAC capability. These structural gaps are a contributing barrier to the provision of evidence-based care. This study shows the need for increased focus and investment in expanding the provision of and improving the quality of these essential, life-saving PAC services.

## Background

Access to safe, legal abortion is limited in Zimbabwe but, despite the restrictive law, an estimated 65,000 abortions occurred in 2016 in Zimbabwe [1]. In Eastern Africa, where Zimbabwe is located, an estimated 76% of abortions are considered unsafe [2]. In Zimbabwe, four in ten women who have an abortion experience complications that require treatment [1], and of the women who do seek treatment for abortion complications, around 40% experienced moderate to severe complications, and 3% were classified as a maternal near-miss [3]. Despite a critical need for post-abortion care (PAC) services to treat abortion complications, there are some indications that the Zimbabwean health system is struggling to provide quality PAC services: over half of facilities that provide PAC in Zimbabwe reported stock-outs of essential PAC medicines and supplies, such as misoprostol and manual vacuum aspiration (MVA) kits [1].

PAC services are essential to improve the health and save the lives of women who experience complications from abortion, particularly unsafe abortions. These services include treatment for complications due to an incomplete abortion or miscarriage to evacuate the uterus as well as provision of post-abortion family planning to prevent future unintended pregnancies. Zimbabwe’s Ministry of Health and Child Care (MoHCC) has made efforts to improve PAC by updating the national PAC guidelines in 2014 [4]. The guidelines include treatment of abortion complications with misoprostol or MVA instead of dilatation and curettage (D&C) for first trimester abortions; task-shifting to mid-level providers; provision of comprehensive post-abortion contraceptive services [4]; and expansion of MVA and misoprostol for uterine evacuation through trainings [5]. However, Zimbabwe has undergone a prolonged period of economic decline and there has been no national assessment of coverage or quality of PAC services [6].

A growing field of scientific literature highlights the importance of focusing on the quality of care in order to make significant impacts on maternal morbidity and mortality [7]. Since abortion is one of the main causes of maternal death, it is essential to understand the coverage and quality of abortion care in a context of high maternal mortality and restrictive abortion laws, like Zimbabwe. While there is a body of literature on measuring the quality of abortion care, there is inconsistency in how the quality of abortion care is measured [8, 9].

There are various components to consider in the measurement of quality of care and the Donabedian quality of care framework, which is used widely in other health sectors, provides a useful framework to evaluate the quality of PAC services in Zimbabwe [10]. In this framework, quality of care encompasses both structure indicators (the setting where care is delivered) and process indicators (the standards of care delivered from provider to patient). The structural component can be measured through signal functions, which are a shortlist of key life-saving interventions that treat abortion complications and measure the capacity and structural quality of PAC [11]. Signal functions were originally developed for emergency obstetric care (EmOC) [11] and were adapted for safe abortion care (SAC) in 2006 [12]. This SAC model was further refined for PAC and termination of pregnancy services in 2016 [13]. The process component of quality of care can be measured through the provision of evidence-based standards of care [9]. The structure and process components allow for the evaluation of both the inputs and standards of care, but do not encompass all aspects of quality, such as women’s experiences of care.

The objective of this paper is to evaluate the structure and process components of quality of PAC services and estimate the population coverage of PAC services within the Zimbabwean health system. We used the signal functions framework to assess structural PAC capacity from a census of PAC-providing facilities and we examined if the process of care is in line with the national PAC guidelines from women’s health records at a sample of these facilities. This approach allows for the unique assessment of gaps between potential capacity to provide quality PAC (based on availability of infrastructure) and actual evidence-based care received by PAC patients (based on documented care processes). No previous studies have examined the Zimbabwean health system’s capacity to provide quality PAC on a national scale [5]. This health systems evaluation of PAC will allow policy makers to identify leverage points to improve coverage and quality of PAC services to reduce abortion-related maternal morbidity and mortality, and researchers can adapt this approach for use in other countries to assess the health system’s capacity to provide PAC.

## Methods

### Data

We used the 2016 Zimbabwe Health Facility Survey (HFS) to estimate the structural capacity of PAC services by calculating the proportion of facilities that can provide basic and comprehensive PAC. The HFS included all public, private and non-governmental (NGO) facilities that had the capacity to provide PAC, which was defined as having an operating theatre or if staff were trained to use misoprostol for PAC per MoHCC guidelines [4]. The list of health facilities came from the MoHCC, the Health Professionals Authority, the Private Hospitals Association of Zimbabwe, the Association of Health Funders of Zimbabwe, and Population Services-Zimbabwe, a local NGO. After removing duplicate facilities, specialized facilities unrelated to PAC, and individual doctors, the study team identified a total of 245 health facilities in Zimbabwe with PAC capacity. After removing 7% of facilities that were deemed ineligible during fielding, a total of 227 facilities participated in the HFS, resulting in a response rate of 100% for all public facilities and a response rate of 97% in private hospitals. More details on HFS sample and study design can be found in Sully et al. [1]. The HFS interviewed providers knowledgeable about PAC service provision at their facility and collected information on current availability and functionality of PAC services and equipment.

We also utilized individual, patient-level clinical data from the Prospective Morbidity Study (PMS) to assess the process of care provided in a nationally representative sample of health facilities. The PMS facilities were selected from the 227 facilities in the HFS, including all central and provincial hospitals (due to expected high PAC caseloads) and a random sample of district hospitals (52%), public primary health centers (30%), private facilities (77%), and NGO facilities (68%). Overall, 127 facilities participated out of the 133 facilities we selected, resulting in a facility-level response rate of 95%. More detail on the PMS sample and study design can be found in Madziyire et al. [3].

The PMS collected information from PAC patients and their providers on complications from spontaneous and induced abortions treated in health facilities in the 28-day study period. All women presenting with incomplete, inevitable, missed, complete, or septic abortion during the study period were eligible for inclusion. We collected information on the medical treatment women received for their complications, as well as receipt of post-abortion contraceptive counseling and services.

All data were collected between August and October 2016. The original study was approved by the Medical Research Council of Zimbabwe, the Joint Research Ethics Committee for the University of Zimbabwe, College of Health Sciences and the Parirenyatwa Group of Hospitals and the Guttmacher Institute’s Institutional Review Board.

### Assessing quality of PAC: structural and process components

We used the HFS for information on the availability of services, supplies and equipment to assess the structural component of quality of care and the PMS for type of service performed to evaluate the process component. For the structural component, we created aggregate indicators of health facilities’ capacity to provide basic or comprehensive PAC utilizing a signal functions approach. While signal functions include some process indicators (such as performing a service like removal of retained products of conception), we utilized signal functions to assess structural capacity, and used the individual-level patient PMS data to better evaluate the process component of quality of care. We used the Campbell et al. adaption of the SAC signal functions model, which includes the essential curative and preventive components of PAC, to evaluate Zimbabwe’s health system’s capacity to provide PAC [12, 13]. Signal functions are divided into two levels of capability: basic and comprehensive. In Zimbabwe, all facilities are expected to have basic PAC capability and referral-level facilities, including public hospitals (district, provincial and central hospitals) and private and NGO facilities, are expected to have comprehensive PAC capability, which include more advanced services to treat severe cases. We did not include the staffing criteria and family planning provision seven days a week as recommended in Campbell et al. because data was not collected on these indicators [13]. The questions on availability and functionality of these signal functions did not specify a time frame. Table 1 presents the six basic signal functions and eight comprehensive signal functions.

**Table 1:** Signal functions for post-abortion care (PAC)

**Basic PAC Signal Functions**	**Comprehensive PACs Signal Functions**
• Perform removal of retained products of conception ^a,b^	• Perform all basic functions (minus communication/referral capacity) plus:
• Administer parenteral antibiotics ^b^	o Provide long-acting reversible contraceptives: implants or IUDs
• Administer uterotonics ^b^	o Perform blood transfusion ^b^
• Administer intravenous fluids ^b^	o Surgical/laparotomy capability ^e^
• Provide contraceptives (condom, pills or injectables) ^c^	
• Communication means or referral capacity ^d^	

^a^ Includes manual/electric vacuum aspiration (MVA/EVA), misoprostol, D&E and dilatation & curettage (D&C) for both ≤12 weeks and> 12 weeks gestation. Although D&C is not a recommended method, the majority of PAC procedures (75%) in Zimbabwe are D&C/D&E [3]. Therefore, we have included D&C as a criteria for removal of retained products to assess capacity

^b^ Facilities were considered to have capability to perform this function if they reported it as a common treatment procedure for their PAC patients in the HFS

^c^ If facilities reported currently having at least one short-acting method out of condoms (male or female), pill or injectables, they were coded as having capability to provide short-acting reversible contraceptive (SARC) methods

^d^ We considered a facility to have referral/communication capacity if they reported commonly having telephone/radio communication for patient services or an ambulance to transport patients to referral facilities

^e^ Facilities that reported an available and functional operating room were coded to have surgical/laparotomy capability

To evaluate the process of care received by PAC patients, we utilized data from women’s health records to create indicators of the key evidence-based standards of care, as specified in the national PAC guidelines, among all women presenting for PAC in Zimbabwe during the study period. These indicators included the use of evidence-based PAC procedure, task shifting to mid-level providers, and providing post-abortion counseling and services. The evidence-based PAC procedures, as recommended by the Zimbabwe MoHCC and WHO, are misoprostol, manual vacuum aspiration (MVA), or electric vacuum aspiration (EVA) for first trimester procedures and dilatation and evacuation (D&E) and misoprostol for second trimester procedures [4, 14].

### Analysis

We calculated the proportion of all facilities that had basic PAC capability and the proportion of referral-level facilities that had comprehensive PAC capability for the structural indicators of quality of care. To assess population coverage of PAC, we estimated the health system’s coverage of basic and comprehensive PAC services based on Zimbabwe’s population size. Population data for 2016 come from the Zimbabwe National Statistic Agency’s Population Projections Thematic Report [15]. The WHO recommends five facilities per 500,000 population with at least one providing comprehensive care for EmOC. This EmOC benchmark recommendation has been used to assess population coverage of abortion services [12, 13, 16]. When analyzing data from women’s health records in the PMS dataset, we applied facility-level weights accounting for the complex sample design, including adjusting for stratification by province and facility level, clustering of women at the facility level, and facility non-response, as well as applying a finite population correction [3]. The statistical analysis was conducted in Stata version 15.0 [17].

## Results

### Structural indicators of quality of PAC

**Fig. 1:**
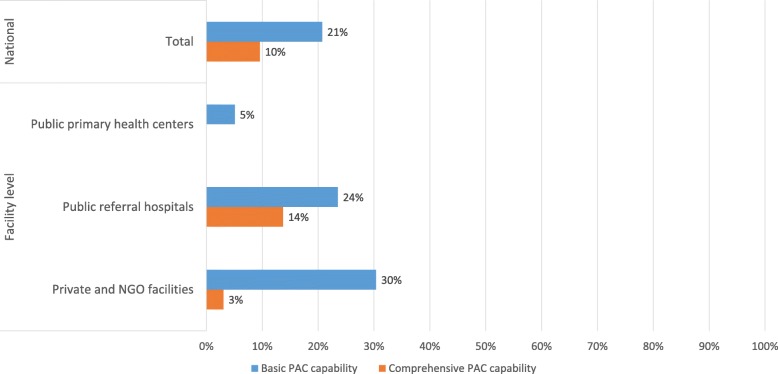
Proportion of facilities with basic and comprehensive PAC capability, nationally and by facility type, Zimbabwe 2016

Nationally, only 21% of all PAC-providing facilities had basic PAC capability, and 10% of referral facilities had comprehensive PAC capability (Fig. 1). Three out of ten private and NGO facilities had basic PAC capability, followed by 24% of public referral hospitals and 5% of public primary health centers. Comprehensive PAC was most commonly offered by public referral hospitals (14%) and only 3% of private and NGO facilities had comprehensive PAC capability (Fig. 1).

Table 2 presents the proportion of facilities performing each basic and comprehensive PAC signal function, which highlights the gaps in the provision of specific essential services that contribute to the overall gaps in PAC capacity. Only around one-third (36%) of public primary health centers had the capacity for removal of retained products of conception. Meanwhile, the majority of public referral hospitals (97%) and private and NGO facilities (89%) had capacity to remove retained products of conception (Table 2). However, public referral hospitals as well as private and NGO facilities often failed to meet basic signal functions due to their inability to provide uterotonics (41 and 47%, respectively) and intravenous fluids (69 and 61%, respectively). For comprehensive signal functions, just over one-third (38%) of all facilities expected to have comprehensive capability (public referral hospitals and private and NGO facilities) were capable of blood transfusions. Most public referral hospitals had surgical/laparotomy capacity (89%) but only 39% of private and NGO facilities had surgical capacity (Table 2).

**Table 2:** Proportion of facilities performing basic and comprehensive PAC signal functions, nationally and facility type, Zimbabwe 2016

	Total	Facility Type		
		Public primary health centers	Public referral hospitals	Private and NGO facilities
**Basic Signal Functions**				
Removal of retained products of conception^1^	79%	36%	97%	89%
Parenteral antibiotics	71%	36%	87%	79%
Uterotonics	44%	46%	41%	47%
Intravenous fluids	61%	49%	69%	61%
Contraceptives (condom, pills or injectables)	87%	97%	86%	79%
Communication means or referral capacity	93%	85%	95%	98%
**Comprehensive Signal Functions**^2^				
Long-acting reversible contraceptives (LARCs): implants or IUDs	81%	–	83%	77%
Blood transfusion	38%	–	49%	21%
Surgical/laparotomy capability	69%	–	89%	39%
**Total number of facilities**	227	59	102	66

^1^ Includes MVA/EVA, misoprostol and D&C/D&E

^2^ Comprehensive facilities must have all of the basic signal functions (excluding referral capacity) plus at least one long-acting reversible contraceptive method (IUDs or implants), blood transfusion and surgical capability. Public primary health centers were not included in the denominator for national comprehensive signal functions

### Process indicators of quality of PAC

Overall, only one out of four PAC patients received the recommended PAC procedure performed with the appropriate technology (Table 3). The proportion of PAC procedures performed with the appropriate technology was slightly less for patients in the second trimester (22%) compared to first trimester procedures (27%). Out of PAC patients who received the appropriate technology, over half (56%) received MVA/EVA and 44% received misoprostol in the first trimester and 61% received dilatation and evacuation (D&E) and 39% received misoprostol in the second trimester (Table 1). Public primary health centers and private and NGO facilities had higher proportions of patients receiving the appropriate technology in the first trimester (30 and 33%, respectively) compared to public referral hospitals (25%) (Table 3). Only 14% of women seen in facilities with comprehensive PAC capability received the appropriate technology in the first trimester, and 38% of women seen in basic PAC capability facilities did. Doctors performed the majority of PAC procedures (91%), except in public primary health centers where nurse midwives performed all procedures (Table 3).

**Table 3:** Process quality of care indicators of post-abortion care based on national PAC guidelines using women’s health records, nationally by facility type and PAC capability, Zimbabwe 2016

Indicator of quality of PAC services	Total		Facility Type			Structural PAC Capability	
	Weighted N^1^	Weighted %	Public primary health centers	Public referral hospitals	Private and NGO facilities	Basic PAC capability	Comprehensive PAC capability
PAC procedure performed with appropriate technology ^2^	260	25%	30%	24%	30%	35%	18%
First trimester^3^	182	27%	30%	25%	33%	38%	14%
Second trimester^4^	77	22%	–	23%	21%	28%	27%
PAC procedures performed by:							
Medical doctor	960	91%	0%	92%	87%	91%	96%
Nurse/midwife/clinical officer	93	9%	100%	8%	13%	9%	4%
Proportion of PAC patients who received contraceptive counseling at discharge^5^	1154	94%	100%	94%	92%	92%	97%
Of PAC patients counseled, the proportion who received modern contraception at discharge^5^	491	43%	61%	39%	61%	42%	34%
Of PAC patients who received modern contraception, the proportion who received:							
Short-acting reversible contraceptive methods^6^	454	92%	100%	94%	82%	91%	94%
Long-acting reversible methods or permanent methods^7^	41	8%	0%	6%	20%	11%	8%
Total number of PAC patients	1302		44	1113	145	400	257

^1^ There were 263 women missing on variable for PAC procedure and an additional 8 missing on the trimester variable; 249 women were missing on PAC provider variable; 72 women were missing on the contraceptive counseling variable

^2^ The WHO recommends misoprostol, manual vacuum aspiration (MVA), or electric vacuum aspiration (EVA) for first trimester procedures and dilatation and evacuation (D&E) and misoprostol for second trimester procedures. The denominator for this calculation is all PAC procedures performed as reported in the PMS

^3^ Of PAC patients who received the recommended first trimester procedure, 51% received MVA, 5% received EVA and 44% received misoprostol (at the national level)

^4^ Of PAC patients who received the recommended second trimester procedure, 39% received misoprostol, 14% received digital evacuation, and 47% received forceps evacuation (at the national level)

^5^ Out of all PAC patients who had been discharged at time of interview (25 PAC patients had not yet been discharged at time of interview)

^6^ Short acting reversible contraceptive methods include male condom, female condom, pills and injectables

^7^ Long acting reversible methods include IUD and implant. Permanent methods include female sterilization (no PAC patients’ partners received male sterilization at discharge). The sum of short acting and long acting methods may exceed 100% since patients may have received multiple methods

Less than half of PAC patients (43%) who were counseled on contraception reported receiving modern contraception (Table 3). Of those who received modern contraception, the majority (92%) received a short acting method. Three out of five PAC patients at public primary health centers (61%) who were counseled received modern contraception and 39% of PAC patients at public referral hospitals who were counseled received modern contraception (Table 3). Only 34% of patients seen in comprehensive PAC capable facilities received modern contraception at discharge (Table 3).

### Population coverage of PAC services

Based on the 2016 total population figure of 14,480,224 [15], Zimbabwe requires 116 facilities with the capability to provide basic PAC services and 29 facilities with comprehensive PAC capability in order to meet the needs of the population. Based on the WHO recommended benchmark and Zimbabwe’s population size, the health system had only 41% of the basic PAC facilities that are recommended for the population, and 55% of the comprehensive PAC facilities that are recommended (Table 4). This translates to 8.3 million Zimbabweans without coverage of basic or comprehensive PAC services, which is 56% of the population (data not shown). The proportion of facilities that met recommended levels varied by province with 17% of facilities meeting basic levels in Bulawayo, Matabeleland South and Mashonaland East and up to 69% in Masvingo. There were no facilities with comprehensive PAC capability in the provinces of Bulawayo, Matabeleland North and Midlands (Table 4).

**Table 4:** Recommended^1^ and actual number of facilities with basic and comprehensive PAC capability, nationally and by province, Zimbabwe 2016

National and provinces	Basic			Comprehensive		
	Recommended	Actual	Proportion of facilities meeting recommend levels	Recommended	Actual	Proportion of facilities meeting recommend levels
National	116	47	41%	29	16	55%
Bulawayo	6	1	17%	1	0	0%
Matabeleland South	6	1	17%	1	1	67%
Mashonaland East	12	2	17%	3	1	34%
Midlands	14	3	21%	4	0	0%
Mashonaland Central	10	3	29%	3	1	39%
Matabeleland North	7	2	30%	2	0	0%
Manicaland	16	8	51%	4	2	51%
Harare	19	10	53%	5	1	21%
Mashonaland West	13	8	60%	3	3	90%
Masvingo	13	9	69%	3	7	215%

^1^ WHO recommends 5 facilities per 500,000 residents, with at least one being comprehensive

## Discussion

This analysis provides the first-ever national evaluation of the coverage and quality of PAC services in Zimbabwe, highlighting the critical gaps in distribution, availability and quality of these essential services. Overall, only one out of five facilities have all of the essential services to provide basic PAC in Zimbabwe. At the population level, the health system has only 41% of the recommended basic PAC facilities and 55% of the recommended comprehensive PAC facilities. The quality of care received by women is also poor with a majority of PAC patients not receiving evidence-based procedures.

The results of low levels of PAC capability are consistent with other national studies evaluating PAC services in Kenya [18], Nepal [19] and Zambia [13]. A multi-country study assessing health system capacity to provide PAC using signal functions also found low levels of capacity in other sub-Saharan African countries. For example, less than 10% of primary-level facilities in Kenya, Rwanda, Tanzania, and Uganda have the capability to provide basic PAC and less than 55% of referral-level facilities in these countries could provide comprehensive PAC [20]. Compared to other PAC signal functions assessments, this analysis allows for more specific insights into the process of care with the use of individual, patient-level data of services received, instead of just the facility-level data of ability to provide PAC services.

Using a health systems approach, this analysis allows us to evaluate PAC capacity by facility level to highlight where essential services are lacking. Just over one-third (36%) of public primary health centers have the capacity to evacuate the uterus, yet they are intended to be the first point of care for women in rural areas, where the majority of the Zimbabwean population lives [21]. Rural PAC patients in Zimbabwe have significantly higher odds of developing higher severity post-abortion complications when compared to urban PAC patients [3]. Uterine evacuation is a safe procedure that can and should be performed at the primary level [9]. Therefore, it is important for public primary health centers to be equipped and providers trained to use appropriate uterine evacuation technologies, such as misoprostol, to avoid delays in treatment associated with referring patients to other facilities. In addition, with only three out of five PAC patients receiving modern contraception at discharge from primary health centers, task-shifting and training of mid-level providers to provide short and long acting contraception in primary health centers could ensure a wide range of choices and increase contraceptive uptake among PAC patients [22, 23]. Our analysis also shows that only 38% of referral-level facilities have the capability to provide blood transfusion services, which is vital for women with hemorrhage and/or anemia, both common post-abortion complications. In addition, IV fluids and antibiotics must be available in all facilities as they form the first line of resuscitation for patients who have experienced severe hemorrhage or sepsis.

In addition, less than half (43%) of PAC patients who received contraceptive counseling also reported receiving a modern contraceptive method. Providing voluntary contraceptive services for PAC patients to prevent future unintended pregnancies is an essential component of PAC. Other studies in Zimbabwe and in the region have found that facilities should have free and comprehensive contraception services within the wards where patients are admitted as going to another unit in the hospital for contraceptive methods is a substantial uptake barrier for many PAC patients [24, 25].

Even if a health facility has the theoretical capacity to provide basic or comprehensive PAC as documented in signal functions, there are still barriers to providing evidence-based care. A study in Malawi found various reasons for low uptake of MVA use and continued preference for curettage (a procedure no longer recommended by the WHO). These included lack of training, supervision, feedback and reliable supplies; health worker’s attitudes towards PAC; and provider’s perceived personal benefits and risks of MVA, such as preferring curettage because they are more familiar and comfortable with it [26]. Ethiopia serves as a successful example of increasing the availability and quality of abortion services after legal reform and major efforts by the Ministry of Health including development and dissemination of evidence-based guidelines; training and task-shifting to midlevel providers; and integrating post-abortion contraception into existing reproductive health services [16]. Interventions to improve the quality of PAC services in Zimbabwe must take a multifaceted and coordinated approach to address the multiple barriers to providing quality, evidence-based care. These could include ensuring sustainable availability of supplies, on-the-job training and supervision [27], and addressing human resources shortages. In addition, the vast majority (91%) of PAC procedures are performed by medical doctors, despite the PAC guidelines for task-shifting to mid-level providers. Other studies have found that increasing communication and teamwork between cadres can improve the quality of care and help with task-shifting [28].

This study has several limitations. The measurement of availability and functionality of PAC signal functions was self-reported from the health provider in the facility, not visually confirmed, and not assessed within a specified time frame of 90 days, as is typically done in signal functions assessments. Therefore, the signal functions levels are most likely an overestimate of capacity. In addition, in the prospective survey of PAC patients, women and their providers were interviewed prior to discharge so it is possible she could have received modern contraception after the interview, and therefore we could have underestimated the proportion of PAC patients receiving modern contraception. The WHO benchmark of one comprehensive EmOC facility per 500,000 residents is likely an underestimate when applied to abortion services since the recommendation is based on the assumption that 15% of pregnancies will result in complications [13]. However, the likelihood of abortion complications, which varies based on the incidence and safety of abortion, may be higher or lower than the risk of obstetric complications. It would be beneficial to re-assess the recommended levels using a benchmark more related to the risk of abortion complications and need for PAC.

## Conclusion

The provision of quality PAC services is an essential component in reducing abortion-related morbidity and mortality, but these results highlight the large gaps in the availability and distribution of facilities with basic and comprehensive PAC capability. Women who seek care for post-abortion complications are also not receiving the evidence-based and recommended standards of care. There is a gap between the commitment to provide PAC and the lack of capacity of the health system to provide these essential services. This study shows the need for increased focus and investment in expanding the provision of and improving the quality of these essential, life-saving PAC services.

## Data Availability

The Health Facilities Survey can be accessed under DOI 10.6084/m9.figshare.5778021, The Prospective Morbidity Survey (PMS) dataset is not publicly available due to confidentiality and privacy concerns. We are determining ethical clearance to make this data set available to other researchers. Please contact Elizabeth Sully (esully@guttmacher.org) to request the data.

## Abbreviations

PAC: Post-abortion care
WHO: World Health Organization
MoHCC: Ministry of Health and Child Care
